# COVID-19 as part of the hyperferritinemic syndromes: the role of iron depletion therapy

**DOI:** 10.1007/s12026-020-09145-5

**Published:** 2020-07-17

**Authors:** Carlo Perricone, Elena Bartoloni, Roberto Bursi, Giacomo Cafaro, Giacomo Maria Guidelli, Yehuda Shoenfeld, Roberto Gerli

**Affiliations:** 1 Rheumatology, Department of Medicine, University of Perugia, Piazzale Giorgio Menghini, 1, 06129 Perugia, Italy; 2 Rheumatology and Clinical Immunology Humanitas Research Hospital, Rozzano, Milan, Italy; 3 Zabludowicz Center for Autoimmune Diseases, Sheba Medical Center, Tel-Aviv University, 5265601 Tel-Hashomer, Israel; 4 The Mosaic of Autoimmunity Project, Saint Petersburg University, Saint Petersburg, Russia; 5 Ministry of Health of the Russian Federation, Sechenov First Moscow State Medical University, Moscow, Russia

**Keywords:** SARS-CoV-2, COVID-19, Anti-viral, Iron, Hyperferritinemic, Hemophagocytic lymphohistiocytosis, Macrophage activation syndrome, Adult-onset Still’s disease, Catastrophic antiphospholipid syndrome, Iron, Deferoxamine, Iron depletion therapy

## Abstract

SARS-CoV-2 infection is characterized by a protean clinical picture that can range from asymptomatic patients to life-threatening conditions. Severe COVID-19 patients often display a severe pulmonary involvement and develop neutrophilia, lymphopenia, and strikingly elevated levels of IL-6. There is an over-exuberant cytokine release with hyperferritinemia leading to the idea that COVID-19 is part of the hyperferritinemic syndrome spectrum. Indeed, very high levels of ferritin can occur in other diseases including hemophagocytic lymphohistiocytosis, macrophage activation syndrome, adult-onset Still’s disease, catastrophic antiphospholipid syndrome and septic shock. Numerous studies have demonstrated the immunomodulatory effects of ferritin and its association with mortality and sustained inflammatory process. High levels of free iron are harmful in tissues, especially through the redox damage that can lead to fibrosis. Iron chelation represents a pillar in the treatment of iron overload. In addition, it was proven to have an anti-viral and anti-fibrotic activity. Herein, we analyse the pathogenic role of ferritin and iron during SARS-CoV-2 infection and propose iron depletion therapy as a novel therapeutic approach in the COVID-19 pandemic.

## Background

The outbreak of the SARS-CoV-2 virus emerged as a pandemic risk in early 2020. The disease (COVID-19) is mainly characterized by fever, dry cough, fatigue and lung involvement leading to pneumonia [1]. Despite most cases having a mild behaviour, up to 14% can be severe with dyspnoea, tachypnea with a respiratory frequency ≥ 30/min, hypoxemia with SpO2 ≤ 93%, partial pressure of arterial oxygen to fraction of inspired oxygen ratio < 300 and/or pulmonary infiltrates involving more than 50% of lung parenchyma within 24 to 48 h. The disease can be life-threating in 5% of cases (i.e. respiratory failure, septic shock and/or multiple organ dysfunction or failure) [2].

So far, no specific treatment has been approved and there is an urgent need for an agent that could either lower the rate of patients entering the critical stage and be lifesaving, especially when acute respiratory distress syndrome (ARDS) occurs. The current treatment strategy includes several anti-viral drugs and anti-rheumatic agents such as chloroquine and hydroxychloroquine that have immunomodulant properties as well as may have a direct anti-viral activity. Nonetheless, a typical hallmark of the disease seems to be a pro-inflammatory condition with markedly high levels of interleukin (IL)-1B, IL-1RA and tumour necrosis factor (TNF)-α in the early phase and higher levels of IL-2, IL-10 and TNF-α in intensive-care-unit patients. Critically ill patients usually develop neutrophilia, lymphopenia and strikingly elevated levels of IL-6 [3].

Indeed, such over-exuberant cytokine release (aka “cytokine storm”) [4, 5] has been already described in SARS-CoV and MERS-CoV pneumonia suggesting that viral load precedes the peak of IL-6 concentration and subsequent radiographic severity [6]. SARS-CoV-2 enters the pulmonary and intestinal cells through the angiotensin-converting enzyme II (ACE2) to infect them [7, 8]. The result of this excessive cytokine release is the infiltration of activated neutrophils into the alveolar space and a fibroproliferative stage leading to interstitial fibrosis [9, 10]. Cytokine release syndrome (CRS) in coronaviruses infection has different causes that have been exemplified by two main mechanisms. The first one is a delayed interferon (IFN) response mediated by multiple structural and non-structural proteins harboured by both SARS-CoV and MERS-CoV that antagonize IFN. The delayed IFN signalling further orchestrates immune responses and sensitizes T cells to apoptosis. The other is the accumulation of inflammatory monocyte-macrophages and neutrophils in the lungs following human coronaviruses infection as demonstrated in both human and animal studies. These cells are the predominant source of cytokines and chemokines associated with the fatal outcome [11]. On these bases, targeted anti-cytokine treatment has been proposed and, in some cases, used successfully. Tocilizumab is a monoclonal antibody directed against an IL-6 receptor. It was developed to treat rheumatoid arthritis (RA) patients as well as CRS as a possible consequence of the administration of chimeric-antigen-receptor-engineered T cell (CAR-T) immunotherapy [12, 13]. Trials to test the efficacy of tocilizumab on severe COVID-19 patients are being carried out in China and Italy [14] (https://www.aifa.gov.it/sperimentazioni-cliniche-covid-19).

Nonetheless, the identification and treatment of hyperinflammation are mandatory. Mehta et al. [15] have recently proposed that COVID-19 can be part of the broader spectrum of hyperinflammatory syndromes characterized by CRS, such as the secondary haemophagocytic lymphohistiocytosis (sHLH) [16]. Notably, one of the cardinal features of these syndromes is hyperferritinemia. Significantly higher ferritin characterizes COVID-19 severity and worse prognosis suggesting that mortality might be due to virally driven hyperinflammation [17]. Circulating ferritin levels may not only reflect an acute phase response but rather play a critical role in inflammation [18]. If moderate levels of hyperferritinemia are associated with autoimmune diseases, including systemic lupus erythematosus, RA, multiple sclerosis and antiphospholipid syndrome (APS) [19–22], typically elevated levels are described in other conditions including macrophage activation syndrome (MAS), adult-onset Still’s disease (AOSD), catastrophic APS (cAPS) and septic shock. In critically ill patients, hyperferritinemia is associated with the severity of the underlying disease [23–25]; moreover, extremely high levels of ferritin (> 3000 ng/ml) seem to be associated with increased mortality in a dose-response manner [26]. Such high levels of ferritin seem to distinguish patients with hemophagocytic lymphohistiocytosis from those with sepsis, septic shock and other conditions in intensive care units (ICUs) and a cutoff of ferritin of 9083 μg/L showed high sensitivity and specificity and may contribute to improved diagnosis of hemophagocytic lymphohistiocytosis in ICU settings [27]. It is very intriguing that a viral infection, specifically Chikungunya, was able to induce a hyperferritinemic syndrome with underlying AOSD and cAPS [28].

## Hyperferritinemic syndrome

The hyperferritinemic syndrome pathogenesis is extremely complex and variable. Genetic mutations, infections, underlying diseases and immunosuppression can play a distinct role in these conditions, leading to the unique epilogue that is hyperferritinemia (> 500 μg/L) and hyperinflammation [29]. According to Schulert et al. [30], despite the numerous protagonists that can play a role in the development of hyperferritinemic syndrome, they might converge in at least two mechanisms that provoke hyperferritinemia: overactivation of T lymphocytes and over-activity of IFN-γ [30]. Nevertheless, recent evidences described the direct role of the H-chain of ferritin in activating macrophages to increase the secretion of inflammatory cytokines [31].

Several diseases that may present both hyperinflammation and hyperferritinemia have been grouped under this common umbrella named hyperferritinemic syndrome (Table 1). These include MAS, a secondary form of HLH, AOSD, cAPS and septic shock [32, 33]. Although these conditions are characterized by different pathogenesis and clinical presentation, it is likely that pathogenically elevated levels of ferritin sustain the inflammatory process [32]. High levels of ferritin are not specific to the abovementioned hyperferritinemic syndrome. Indeed, levelling up to 2000 μg/L of ferritin can be found in other conditions such as liver damage and infections, the first being one of the possible clinical manifestations of HLH while the second the most common trigger of secondary HLH [34]. Nonetheless, as abovementioned, very high levels can identify patients with HLH in ICU settings [26, 35].

**Table 1:** The spectrum of hyperferritinemic syndromes: suspected aetiologies, clinical features and therapeutic strategies

Hyperferritinemic syndromes			
Name	Aetiology	Clinical features	Therapeutic strategy
Secondary haemophagocytic lymphohistiocytosis	Infections • Viruses • Bacteria • Parasites • Fungi Malignancies • Mainly malignant lymphoma Autoinflammatory or autoimmune disorders Other causes • Transplantation • Metabolic • Traumatic • Iatrogenic (immunosuppression, vaccination, surgery, haemodialysis) • Pregnancy	Fever, rash, hepatosplenomegaly, lymph node enlargement, bleeding diathesis, sepsis-like syndrome, variable degrees of neurologic symptoms, possibly rapidly unexpected progress to multiple organ failure	HLH-94 protocol: • Glucocorticoids • Cyclosporine A • Intrathecal therapy • Etoposide Treatment of the specific trigger/underlying disease: • Glucocorticoids • Anti-viral drugs • Anti-CD20 (rituximab) • Intravenous immunoglobulins • Chemotherapy • IL-1 inhibitors (anakinra, canakinumab) • IL6 inhibitors (tocilizumab) Currently being tested: • JAK1/2 inhibitors (ruxolitinib) • anti–IFN-γ (alemtuzumab, emapalumab)
Catastrophic antiphospholipid syndrome	Trigger supposed to be infections in the presence of antiphospholipid antibodies	Microvascular thrombosis: renal insufficiency, acute respiratory distress syndrome/pulmonary embolism, encephalopathy, stroke, seizures, headache and coma, heart failure, myocardial infarction, valvular defects, livedo reticularis, skin necrosis and digital ischemia; spleen, adrenal glands, pancreas, retina and bone marrow infarction	Intravenous heparin Glucocorticoids Intravenous immunoglobulins Cyclophosphamide Anti-CD20 (rituximab) Plasmapheresis Eculizumab
Adult onset Still’s disease	Not clearly defined • Viruses • Bacteria • Solid cancers • Haematological malignancies	Fever, arthritis, skin rash, myalgias, splenomegaly, lymphadenopathy, sore throat, liver involvement, pleurisy or pericarditis, abdominal pain, aseptic meningitis, disseminated intravascular coagulation, haemolysis	Glucocorticoids Hydroxychloroquine Intravenous immunoglobulins Methotrexate Cyclosporine IL-1 inhibitors (anakinra, canakinumab, rilonacept) IL-6 inhibitors (tocilizumab) TNF-inhibitors (infliximab, etanercept and adalimumab)
Septic shock	Infections • Viruses • Bacteria • Parasites • Fungi	Fever, rash, disseminated intravascular coagulation, variable degrees of neurologic symptoms, possibly rapidly unexpected progress to multiple organ failure	Broad spectrum antibiotic therapy Fluid resuscitation Vasopressors

### Hemophagocytic lymphohistiocytosis

HLH (also called hemophagocytic syndrome - despite this term is now outdated [36]) is a rare but potentially life-threatening aberrant hyperferritinemic condition [29]. In a retrospective analysis, the 30-day mortality from clinical onset was 35% (45/129) in young patients and 58% (44/76) in patients older than 60 years [37]. In adults, the clinical characteristics of HLH include fever, rash, hepatosplenomegaly, lymph node enlargement, potential bleeding diathesis, sepsis-like syndrome with or without variable degrees of neurologic symptoms and a possibly rapidly unexpected progress to multiple organ failure [38]. Hyperferritinemia, liver dysfunction, cytopenia, hypertriglyceridemia, hypofibrinogenemia, elevated D-dimer and lactate dehydrogenase are frequently observed [29]. Interestingly, in a large single-centre case series, very high levels of ferritin (> 50,000 μg/L) correlated with 30-day mortality [39], and the drop in ferritin level due to the treatment could have an important prognostic value [16].

As reported in the 2019 HLH recommendation, primary and secondary HLH, including MAS-HLH, have a common terminal pathway but with different pathogenic roots [36]. The primary or familial form (FHLH) begins at an earlier age and tends to be more aggressive. It is due to different gene mutations (PRF1, UNC13-4, STX11, STXBP2, etc.) that lead to the dysregulation of the inflammasome [40, 41] and/or to the reduction in cytotoxic activity of T cytotoxic lymphocytes (CTL) and natural killer cells (NK); degranulation and the control of macrophages or cell apoptosis can be impaired [42, 43]. Cytotoxic deficiency can lead to persistent antigen exposure of lymphocytes, inducing an overproduction of various inflammatory cytokines, in particular IFN-γ, and consequently to CRS and uncontrolled activation of macrophages [44].

The secondary form (sHLH) can occur in different conditions among which viral infections are amid the most frequent. Other infections include bacterial [45], parasitic and fungal [46, 47]. Solid or blood malignancies represent other possible causes (40 to 70% of HLH cases in adults) followed by systemic autoinflammation and autoimmune diseases, in which case sHLH is usually named MAS-HLH [48]. Several rheumatologic diseases can develop MAS-HLH, such as systemic lupus erythematosus, RA, Sjögren’s syndrome, vasculitis and, most frequently, systemic juvenile idiopathic arthritis (sJIA), AOSD and cAPS. Finally, conditions of acquired immune deficiency occurring, for instance, after organ transplantation are rarer triggers of sHLH [36].

Virus infections are the main cause of sHLH, especially the Epstein Barr virus (EBV) [49], Herpes simplex virus (HSV) and cytomegalovirus (CMV). How these viral agents are able to trigger HLH is not fully understood. It seems that they may suppress CTL and NK cell cytotoxicity, predisposing to the development of HLH. EBV latent membrane protein-1 (LMP1) can transcriptionally inhibit lymphocyte activation molecule (SLAM)–associated protein (SAP) leading to overt T cell activation and cytokine production, especially IFN-γ [50]. Recombinant hemagglutinin (H5) from H5N1, causing agent of avian influenza, may suppress the perforin expression and reduce cytotoxicity of human CTL in vitro. At the same time, it promotes an overproduction of IFN-γ that may play an important role in macrophages overactivation, cytokine storm and hemophagocytosis—all features observed in severe H5N1-infected patients [51]. In addition, H1N1 influenza, directly infecting NK cells, reduced their number and their activity [52].

Nevertheless, these mechanisms cannot always explain the development of HLH. Infectious triggers are not always identified, and defects in cytotoxic CTL may not be present [44]. In other HLH models, a prominent role seems to be played directly by the innate immune pathways instead of the CTL and NK activity [44] with the production of IL-1 family cytokines, especially IL-18 and IL-33 [30, 53]. Beside the main treatment of HLH based on HLH-94 protocol, consisting corticosteroids, cyclosporine A, intrathecal therapy and etoposide [36, 54], the treatment of the specific trigger is essential because of the vast heterogeneity of the aetiology of HLH in adult patients. Sometimes, the specific treatment of the trigger agent can be able to control the HLH syndrome without the need of the HLH-94 protocol, as in the case of autoimmune diseases including SLE [55]. Interesting trials testing alternative therapeutic approaches have been promoted, such as those incorporating ruxolitinib (JAK1/2 inhibitor; ClinicalTrials.gov identifiers NCT02400463, NCT03795909, NCT03533790), anakinra (IL-1 blockade; NCT02780583), alemtuzumab (NCT02472054) and emapalumab (anti–IFN-g monoclonal antibody; NCT01818492).

### Catastrophic antiphospholipid syndrome

cAPS is characterized by microthromboses involving at least three organs within a week and is a rare but severe complication of APS. It affects about 1% of APS patients and the mortality rate reaches 36%. Agmon-Levin et al. demonstrated that hyperferritinemia can be found in patients with primary APS and that it correlates with cAPS (71% of cAPS had hyperferritinemia) [56]. Similarly, in SLE patients, hyperferritinemia correlates with thrombocytopenia, the presence of lupus anticoagulant and anti-cardiolipin antibodies, suggesting it could be an early marker for secondary APS [57]. An emerging complication occurring in COVID-19 is coagulopathy and possible thrombotic microangiopathy [58]. A case of COVID-19 and antiphospholipid antibodies with multiple infarcts has been recently described. Interestingly, markedly elevated ferritin was found also in this patient, strikingly reinforcing the connection between infection, coagulopathy and hyperferritinemia [59].

### Septic shock

According to the last International Consensus Definitions for Sepsis and Septic Shock, sepsis is defined as life-threatening organ dysfunction that can be represented by an increase in the Sequential Sepsis-related Organ Failure Assessment score of 2 points or more, caused by a dysregulated host response to infection [60]. Sepsis can be a life-threatening condition and sometimes can have features in common with HLH, such as hyperferritinemia. As reported by the 2019 guidelines of HLH, forms of sepsis characterized by a marked inflammation, but less than a proper form of HLH, may not fulfil the diagnostic criteria of HLH and are described as ‘MAS-like’. For this reason, in critically ill patients with a confirmed or presumed case of sepsis, it is important to exclude the diagnosis of HLH [61]. It is important to underline that viremia, as it has been demonstrated for DNAemia due to HSV type 1, human herpesvirus 6, EBV, CMV and adenovirus, was associated with hyperferritinemia and adverse outcome in paediatric severe sepsis [62]. Whether there is a correlation between SARS-CoV-2 replication and ferritinemia would be of great interest.

## Ferritin in inflammation and viral diseases

Ferritin serves to bind iron molecules and to store iron in a biologically available form for vital cellular processes while protecting proteins, lipids and DNA from the potential toxicity of this metal element. It has been shown that ferritin is composed of two isoforms: H- and L-, differently enriched ferritin is expressed in several tissues [63] and have different implications during inflammation [31]. Ferritin and its subunits light chain ferritin (LHC) and heavy chain ferritin (HFC) showed in vivo and in vitro immunomodulatory effects [64]. For example, HFC in vitro directly binds chemokine receptor 4 (CXCR4) and affects CXCR2-mediated ERK1/2 activation [65]. Despite the acute rise of blood value of ferritin as part of the normal systemic response to inflammation, a hyperferritinemic response is associated with a significantly increased mortality in septic patients [24, 27]. Although the main modulator of ferritin levels is iron availability, its synthesis may also be regulated by different inflammatory cytokines such as IL-1β and IL-6 [66, 67]. Indeed, serum ferritin is affected by upregulation of hepcidin whose production, in turn, is stimulated by pro-inflammatory cytokines, particularly IL-6 [68]. Ten Kate et al. found that in patients with AOSD the amount of iron bound to ferritin is significantly lower compared with samples from healthy controls and patients with hemochromatosis; however, the total amount of circulating iron is much higher than in controls. This suggests that in active AOSD the rapid synthesis of ferritin exceeds the rate of iron incorporation in ferritin [69].

Besides ferritin, another aspect to be considered in viral infections is the impact of iron overload. Iron is required for viral replication and other processes including mitochondrial function, ATP generation, DNA/RNA synthesis and repair and cell survival/ferroptosis [70]. For instance, the activity of the helicases of the SARS-CoV for viral replication requires ATP hydrolysis that in turn needs the presence of iron [71]. Iron overload leads to a worse prognosis in HBV and HCV viral infections and iron supplementation increases the mortality in HIV patients, irrespectively of the anaemic status [72–75]. It is likely that SARS-CoV-2 requires iron for viral replication and for its functions, and this is among the rationales for iron chelation therapy in COVID-19 [76].

Furthermore, iron has an effect on the regulation of T lymphocyte sensitivity to the IFN-γ/STAT1 signalling pathway. Indeed, it is known that the refractoriness of T cells to the IFN-γ/STAT1 pathway has been attributed mainly to downregulation of the IFN-γR chains, especially IFN-γR2. In human T lymphocytes, IFN-γR2 internalization occurs mostly in clathrin-coated pits independently from IFN-γ [77]. Iron binds to cytoplasmic iron regulatory protein 1 (IRP1) and IRP2 which, in turn, regulates expression of proteins such as ferritin. In addition, there is a regulatory loop connecting nitric oxide (NO) and iron: on the one hand, NO modulates IRP activity [78, 79], and, on the other hand, iron impairs inducible NO synthase transcription. It was shown that iron is critical to determine IFN-γR2 internalization thus preventing the activation of the IFN-γ/STAT1 pathway in human T cells. Deferoxamine (DFO), a common iron chelator, can induce an upregulation of IFN-γR2 expression on the cell surface only in activated T cells that have entered the cell cycle [80]. This can restore T cell response to SARS-CoV-2 infection in two ways: (a) restoring the sensitivity of T lymphocytes to IFN-γ, (b) possibly inhibiting clathrin-mediated SARS-CoV-2 cell entry [81].

Nonetheless, Merad and Martin [82] recently addressed the potentially pathological roles of monocytes and macrophages during SARS-CoV-2 infection. Infiltrating macrophages seem to promote acute inflammation and are involved in fibrotic complications observed in patients under mechanical ventilation. NLRP3 inflammasome activation is likely to occur during the infection and viral accessory protein are potent activators of pro-IL-1β gene transcription and protein maturation. Microvascular thrombotic involvement appears to be mediated by activated monocytes through the production of tissue factor and activation of the extrinsic coagulation pathway. Neutrophils recruitment by activated endothelial cells and release of neutrophil extracellular traps (NETs), in turn, further amplify the coagulation process. The early recognition of such a massive inflammatory process resembling that of MAS-HLH remains a diagnostic challenge and research focused on the potential diagnostic application of specific biomarkers apart from ferritin [83]. Soluble (s) CD163 is the soluble counterpart of the CD163 receptor for the haemoglobin–haptoglobin complex located on M2 macrophage cell membrane and represents a marker of M2 macrophage activation and differentiation. The shedding and quick release of CD163 is induced by several pro-inflammatory stimuli such as TNF-α, oxidative stress and lipopolysaccharide and several studies demonstrated the prognostic role of sCD163 in conditions characterized by a high systemic inflammatory burden, like sepsis or acute respiratory distress syndrome requiring mechanical ventilation [84, 85]. Moreover, convincing evidence supports the potential role of sCD163 as a biomarker of MAS-HLH [86, 87]. In this setting, activated or hemophagocytic CD163+ macrophages within bone marrow aspirates were demonstrated to precede MAS-HLH development in subjects with sJIA, thus suggesting the pivotal role of macrophage activation in MAS-HLH through the induction of hemophagocytosis and hypercytokinemia [88]. Of note, serum sCD163 levels are significantly increased in patients with sJIA associated with MAS-HLH in comparison to patients with an active disease without MAS-HLH, in particular at disease onset, follow the clinical course in response to treatment and, of note, correlate with other surrogate biomarkers of systemic inflammatory burden, like sCD125 and ferritin [89]. Similar increases of sCD163 have been depicted in patients with AOSD, in particular in the group with active disease, and levels were similar for patients with sepsis [90]. Interestingly, sCD163 levels positively correlated with ferritin serum levels only in AOSD patients, suggesting a direct involvement of macrophage in ferritin production in these conditions [91]. Moreover, a recent study demonstrated that systemic lupus erythematosus patients with MAS-HLH display significantly higher levels of sCD163 in comparison with patients with other severe disease manifestations like lupus nephritis, autoimmune haemolytic anaemia or immune thrombocytopenia, with sCD163 levels correlating with disease activity [91]. Levels of sCD163 may also serve as markers to differentiate primary HLH and MAS-HLH. In a recent study, the serum levels of sCD163 were markedly increased in patients with MAS-HLH as compared with pHLH patients, thus hypothesising that the macrophage activation in MAS-HLH is higher than in pHLH [92]. Thus, in MAS-HLH, the massive IL-1β release triggers a close autocrine loop leading to cytokine storm with dramatic IL-6, IL-18 and ferritin production and, consequently, sCD163 spreading from macrophages. Surely, a deeper understanding of this complex pathogenic pattern related to massive cytokine release may lead to targeted therapies and improved patient prognosis [16].

## Iron depletion therapy

As a consequence of the abovementioned pathogenic scenario linking iron, inflammation and infections, there is the need to find a possible therapeutic strategy to prevent CRS and onset of fibrosis occurring particularly in patients with COVID-19. The progress in understanding the critical role of pro-inflammatory cytokines in the pathogenesis of other hyperferritinemic syndromes such as MAS-HLH and AOSD has led to pilot the use of anti-cytokine agents, resulting in an increasing number of successful case reports in patients who were unresponsive to conventional treatments [93]. The inhibition of IL-1 (with the use of anakinra and canakinumab) and IL-6 (mainly with tocilizumab) showed a strong efficacy compared with placebo in several cohorts and randomized controlled trials in MAS-HLH and AOSD. In a post hoc analysis of data from MEASURE, a randomized, multicentre, double-blind, 24-week, phase 3B trial of tocilizumab in RA, authors depicted a rapid decrease of ferritin, hepcidin and haptoglobin following tocilizumab administration. This is consistent with the idea that IL-6 signalling is a common stimulus to the production of these molecules [94, 95]. The anti-IL-6 effect on ferritin could explain part of the emerging successful reports on tocilizumab treatment in SARS-CoV-2 infection.

**Fig. 1:**
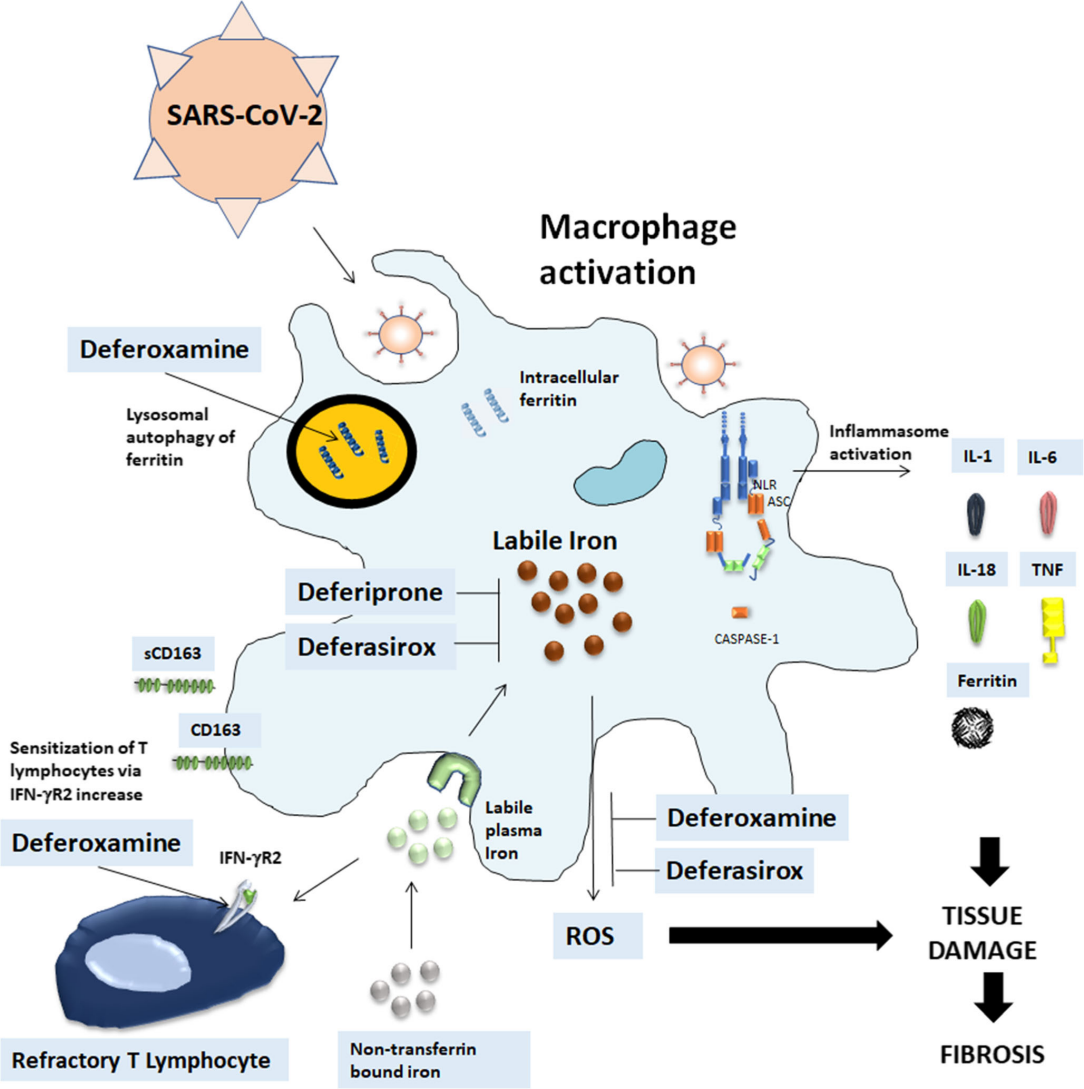
Iron chelation therapy in SARS-CoV-2 infection. SARS-CoV-2, likely through inflammasome activation, leads to stimulation of infiltrating macrophages that can promote hyperinflammation, characterized by increased levels of IL-6, TNF-α, IL-1β, ferritin and subsequent possible lung fibrotic complications. The increased ferritin production allows adequate storage of iron and deprives the pathogen of iron. Labile iron in the cell contributes to the formation of reactive oxygen species that further promote tissue damage and fibrosis. Iron accumulates in the reticuloendothelial macrophages and the shedding of CD163 is the marker of macrophage activation. Iron chelation therapy can interrupt these steps. (**a**) Deferoxamine (DFO) has a direct effect on ferritin since promotes ferritin degradation in lysosomes by inducing autophagy. Both deferiprone and deferasirox are likely to chelate cytosolic iron and iron which is extracted from ferritin prior to ferritin degradation by proteasomes. (**b**) DFO can induce an up-regulation of IFN-γR2 expression on the cell surface on activated T cells thus restoring T cell response to SARS-CoV-2 infection. (**c**) Deferasirox and DFO reduce fibrosis-inhibiting the production of free radicals, macrophage tissue infiltration and cause a remarkable decrease of IL-6 levels

Nonetheless, the rapidity of the onset of inflammation in the acute phase of SARS-CoV-2 infection may provoke increased ferritin production to permit adequate storage of iron and to deprive the pathogen of iron. If the binding capacity of transferrin in the blood is exceeded, iron may be found in the plasma as non-transferrin bound iron that changes to its redox-active form termed labile plasma iron (LPI) [96]. LPI correlates with ferritin levels and contributes to the formation of reactive oxygen species (ROS) resulting in tissue damage and subsequent fibrosis [97] (Fig. 1). Thus, a novel approach to COVID-19 treatment can be represented by iron chelation therapy that can interrupt these steps. Iron chelation represents a pillar in the treatment of iron overload due to a wide spectrum of diseases and multiple chelating agents are currently registered and routinely used in clinical practice. Indeed, deferoxamine (DFO) has a direct effect on ferritin since promotes ferritin degradation in lysosomes by inducing autophagy, while both deferiprone and deferasirox are likely to chelate cytosolic iron and iron which is extracted from ferritin prior to ferritin degradation by proteasomes [98] (Fig. 1). Moreover, several studies have been performed on the potential anti-viral effect of iron-chelating therapy. Indeed, iron overload can contribute to HIV replication in vitro by increasing reverse transcriptase activity and reducing the viability of infected T cells. Iron chelation by DFO has shown beneficial effects on HIV infection [99] probably through multiple mechanisms such as (1) restriction of DNA synthesis through the inhibition of ribonucleotide reductase, which requires iron to exert its enzymatic activity, (2) inhibition of T cell proliferation that is essential for HIV replication, (3) direct toxic effect on viral DNA and RNA via oxidative stress and (4) inhibition of NF-kB pathway. These effects may not be universal for all iron chelating agents. In fact, DFO and deferiprone (DFP) can both inhibit T cell proliferation and DNA synthesis, while bleomycin can directly bind to viral DNA with no effect on host T cells [100, 101].

A potential anti-viral effect has also been demonstrated with other pathogens, such as HSV-1 [102] and CMV. More specifically, CMV requires iron in order to induce an increase in the size of infected cells, so that increased in vitro levels of free iron have been demonstrated before the occurrence of this phenomenon, which can be effectively limited by iron chelation therapy [103]. DFO is also capable to further enhance the therapeutic effect of IFN on hepatitis B virus (HBV) infection [104]. Fewer data are available on the effect of iron chelation on other infective agents, though Mateos et al. [105] reported increased levels of free iron in the bronchoalveolar lavage (BAL) fluid of HIV patients with *Pneumocystis jiroveci* pneumonia compared with controls, suggesting a potential pathogenic role of iron. Similarly, a beneficial effect of DFO treatment was demonstrated in a murine model of *Trypanosoma cruzi* infection, independently from the iron metabolism of the host cell [106]. However, it should be carefully considered that iron chelators may actually be exploited by pathogens as sources of iron [107], thus a careful analysis of the pharmacodynamic mechanisms of the single chelating agents available is warranted.

One of the main mechanisms through which iron can promote inflammation is mediated by increased production of free oxygen radicals via the Haber-Weiss reaction. As an example, iron is able to increase the in vitro production of IL-6 by endothelial cells following infection with *Chlamydia pneumoniae* and influenza A virus, which can be effectively controlled by DFO [108]. Interestingly, similar processes, including IL-6 and free oxygen radical production, take place during septic shock. Thus, it is not surprising that iron chelation is effective in decreasing mortality in murine models of septic shock via NO scavenging [109] and inhibition of MAP kinases and NF-kB pathways, eventually leading to reduced production of pro-inflammatory cytokines [110].

One of the most severe complications of diseases leading to iron overload is liver damage, characterized by progressive fibrosis and, eventually, irreversible cirrhosis. In fact, the prevention of liver damage is the main indication of iron chelation in these conditions. Although the reduction of free iron levels and, consequently, of oxygen radicals, is the main mechanism preventing progressive damage, some authors suggested that iron-chelating agents may exert an independent anti-fibrotic effect. This evidence comes from studies showing a reduction of liver fibrosis in the absence of a significant decline in liver iron content [111]. Deferasirox (DFX) and DFO seem able to reduce damage and fibrosis in multiple rat models of concavalin A and CCl_4_-induced liver injury by inhibiting the production of free radicals [112–114], though other studies did not confirm this evidence [115]. Anti-fibrotic effects in kidney disease have also been demonstrated in rat and mouse models of renal damage, again via a reduction of oxidative stress, macrophage tissue infiltration and production of pro-fibrotic cytokines such as TGF-β [116, 117]. Other authors showed that DFO can provoke a remarkable decrease in IL-6 levels and have a potent anti-fibrotic effect in HCV infection [114].

## Conclusions

Whether these phenomena share common aspects with COVID-19 is currently not known. It is, however, reasonable to speculate that iron chelation may influence free radicals and pro-inflammatory cytokines production that is strongly involved in the late phase of COVID-19, eventually leading to acute lung injury and ARDS. It has been shown that mechanical ventilation, often required in COVID-19 patients, may induce lung injury that is known to be associated with the release of inflammatory factors, apoptosis, endothelial dysfunction and activation of the coagulation system [118, 119]. Interestingly, pre-conditioning with DFO showed a lung-protective effect against mechanical ventilation through effective reduction of ROS formation in macrophages and mitochondria in a mouse model [120].

Additionally, preliminary data seem to suggest that residual lung damage may be present in a subset of severe COVID-19 patients following the acute phase of the disease [121]. If these data were to be confirmed, the anti-fibrotic effect of iron chelating agents may represent an additional mechanism of action deserving careful consideration. There are so far two trials to evaluate the efficacy and safety of deferoxamine in patients with COVID-19 (NCT04333550, NCT04361032) either compared with standard treatment or to tocilizumab, results are eagerly awaited.

To conclude, the abovementioned considerations lead to the idea that COVID-19 may be part of the hyperferritinemic syndrome spectrum [122]. Possible iron acute overload caused by rapid synthesis of ferritin exceeding its iron incorporation rate and the beneficial effects of iron chelation therapy on the inflammatory status as well as on the fibrogenesis occurring in the lungs suggest that, in appropriate setting of critically ill patients with COVID-19, iron chelation therapy could be considered to improve survival and overall long-term outcome.
